# Experience-dependent plasticity in the olfactory system of *Drosophila melanogaster* and other insects

**DOI:** 10.3389/fncel.2023.1130091

**Published:** 2023-02-22

**Authors:** Benjamin Fabian, Silke Sachse

**Affiliations:** Research Group Olfactory Coding, Max Planck Institute for Chemical Ecology, Jena, Germany

**Keywords:** olfaction, plasticity, odor experience, *Drosophila*, insects, antennal lobe

## Abstract

It is long known that the nervous system of vertebrates can be shaped by internal and external factors. On the other hand, the nervous system of insects was long assumed to be stereotypic, although evidence for plasticity effects accumulated for several decades. To cover the topic comprehensively, this review recapitulates the establishment of the term “plasticity” in neuroscience and introduces its original meaning. We describe the basic composition of the insect olfactory system using *Drosophila melanogaster* as a representative example and outline experience-dependent plasticity effects observed in this part of the brain in a variety of insects, including hymenopterans, lepidopterans, locusts, and flies. In particular, we highlight recent advances in the study of experience-dependent plasticity effects in the olfactory system of *D. melanogaster*, as it is the most accessible olfactory system of all insect species due to the genetic tools available. The partly contradictory results demonstrate that morphological, physiological and behavioral changes in response to long-term olfactory stimulation are more complex than previously thought. Different molecular mechanisms leading to these changes were unveiled in the past and are likely responsible for this complexity. We discuss common problems in the study of experience-dependent plasticity, ways to overcome them, and future directions in this area of research. In addition, we critically examine the transferability of laboratory data to natural systems to address the topic as holistically as possible. As a mechanism that allows organisms to adapt to new environmental conditions, experience-dependent plasticity contributes to an animal’s resilience and is therefore a crucial topic for future research, especially in an era of rapid environmental changes.

## 1. Introduction

The word “plasticity” is exceptionally popular nowadays and is used to describe various phenomena in different branches of science, particularly in neuroscience. Searching for this term in scientific texts yields about 3 million hits, a similar number to other commonly used words such as “neuron” or “*Drosophila*,” which yield 3.3 million and 2 million hits, respectively, and it generates even half as many hits as “climate” (*via* Google Scholar, as of 03.11.2022). Although the term is used so frequently, it is inadequately defined, probably largely due to ignorance of its original meaning.

In the 1860s, at the beginning of the industrial revolution, the concept of plasticity originated in physics and material sciences (Tresca, 1864). At this time, a hypothesis about a peculiar property of metals was published by Henri Tresca. According to his hypothesis, metals enter a flowing state when an applied force exceeds a certain threshold. This work formed the basis for our current comprehension of the term “plasticity” in the physical sense, according to which solid bodies deform in response to an applied force and retain this state even after the force is removed (Bruhns, 2018).

It only needed a few decades for this term to find its way into biology. William James used the term for the first time in a surprisingly modern way (Berlucchi and Buchtel, 2008) to link the morphological plasticity of organisms to their behaviors (James, 1890). He suggested that the observed behaviors of organisms depend on the plasticity of the organic matter that constitutes the organisms. He already emphasized that structural plasticity needs not necessarily to involve only the visible morphology of an organism, but can also take place at the molecular level. Moreover, James highlighted nervous tissue as an organic matter with an exceptional amount of plasticity. It was his adoption of the term “plasticity” that bridged the gap between biology and physics and that later became accepted in neuroscience.

Demoor (1896), Ramón y Cajal (1894), Lugaro (1906, 1909), and Minea (1909) were the first neuroscientists to demonstrably use the term “plasticity” (DeFelipe, 2006; Mateos-Aparicio and Rodríguez-Moreno, 2019). However, it took several decades for the term to become as popular as it is today. Shortly after 1970, the terms “plasticity” and “neuroplasticity” were used by an increasing number of neuroscientists. As early as 1976, it was noted that the term was being used more and more broadly without being properly defined (Paillard, 1976). Paillard proposed that plasticity describes the ability of a system to acquire new functions by transforming its internal connectivity or by changing the elements of which it consists in response to the internal or external environment (Berlucchi and Buchtel, 2008; Will et al., 2008). According to this definition, a phenomenon can be called plastic only if it combines a morphological and functional change. In addition, the changes must be lasting, even if the event that triggers the effect is only temporary. If morphological and functional changes are reversible, the term “elasticity” would be more appropriate. Since Paillard’s views were published, the term’s popularity has only grown, and has been paralleled by an intensified decline in the accuracy of its use (Jones, 2000; Will et al., 2008; Groh and Meinertzhagen, 2010), which today reached a state where “plasticity” is often just a synonym for “change” or “difference.”

Morphological and functional changes were observed in various parts of the nervous system of insects, including motor neurons and the visual system (Sugie et al., 2018). In this review, we focus on morphological and physiological changes in the insect olfactory system in response to environmental stimuli and on associated behavioral changes. To this end, we first present the composition of the olfactory circuitry of *Drosophila melanogaster* Meigen, 1830 as an example of a well-studied olfactory system and then show how plasticity affects this system in different insect species, emphasizing on recent discoveries in *D. melanogaster*. Various molecular mechanisms that give rise to plasticity effects are highlighted. We address challenges in this research area and suggest how they can be overcome. Furthermore, the transferability of plasticity effects observed in the laboratory to more natural conditions is discussed. Finally, we argue that neuronal plasticity may be a rapid and efficient way to enable organisms to adapt to new environmental conditions, a capability that is of paramount importance in the face of environmental degradation and climate change. With this review, we aim to shed light on this area of research by providing the most comprehensive overview to date on experience-dependent plasticity in the olfactory system of insects.

## 2. The olfactory system of *Drosophila melanogaster*

### 2.1. Olfactory organs, receptors, and sensory neurons

**FIGURE 1:**
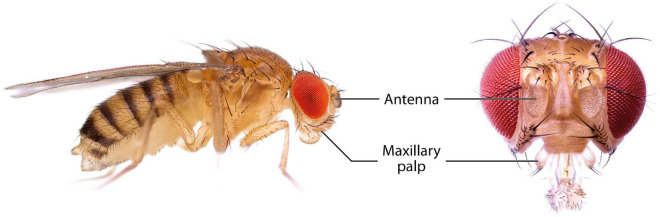
Photographs of *D. melanogaster* showing the main olfactory organs, the antennae and the maxillary palps.

**FIGURE 2:**
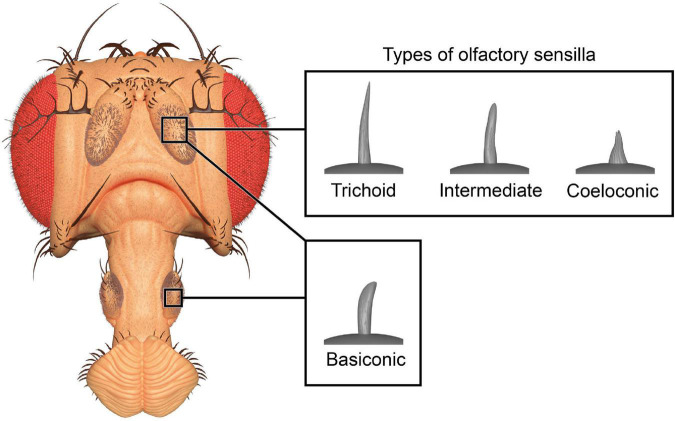
Three-dimensional render of olfactory sensilla types. Club-shaped basiconic sensilla are found on antennae and maxillary palps, while hair-like trichoid, cone-shaped coeloconic, and intermediate sensilla are only found on the antennae. The 3D model of the head is based on photographs and the models of sensilla are based on published micrographs (de Bruyne et al., 2001; Yao et al., 2005).

The olfactory system of *D. melanogaster* is one of the experimentally most accessible parts of any insect brain and therefore an ideal model system for the investigation of plasticity effects. Like other insects, the vinegar fly detects odorants with its antennae, more specifically the third antennal segment (the funiculus), and the maxillary palps (Figure 1; Hansson and Stensmyr, 2011). The cuticular surface of these structures is covered with various types of sensilla, some of which have an olfactory function while others are involved in the perception of non-olfactory cues. There are about 410–460 olfactory sensilla on the funiculus and 60 on the maxillary palp with slight differences between sexes. The olfactory sensilla can be morphologically categorized into four groups: club-shaped basiconic, spine-shaped trichoid, cone-shaped coeloconic, and morphologically less well-defined intermediate sensilla, which combine characteristics of basiconic and trichoid sensilla (Figure 2; Stocker, 1994; Shanbhag et al., 1999; Laissue and Vosshall, 2008; Galizia and Sachse, 2010). These different types are distributed in a stereotypic manner across the funicular surface. The cuticular surface of olfactory sensilla is covered with small pores, which allow volatile compounds to enter (Steinbrecht, 1997), but prevent the sensillar lymph from exiting. The transition from air to an aqueous medium poses a problem for volatile compounds, especially if they are hydrophobic. Odorant-binding proteins (OBPs) are present in high concentration in the aqueous sensillar lymph of sensilla and are thought to facilitate the transition, to transport compounds to the receptors of olfactory sensory neurons (OSNs) (Steinbrecht, 1998; Pelosi et al., 2006; Leal, 2013) and/or to contribute in the activation of receptors by forming a complex with the odorant (Laughlin et al., 2008). OBPs are also hypothesized to play a role in clearance of compounds from receptors to terminate responses (Vogt and Riddiford, 1981; Ziegelberger, 1995; Scheuermann and Smith, 2019). The chemoreceptors are anchored in the dendritic membrane of OSNs (Benton, 2006). OSNs express olfactory (ORs), gustatory (GRs), or ionotropic (IRs) receptors (Benton, 2006; Joseph and Carlson, 2015). Each sensillum houses dendrites of 1–4 OSNs in defined combinations (de Bruyne et al., 2001). Each OSN type expresses one or only very few chemoreceptors each (Fishilevich and Vosshall, 2005; Martin et al., 2013). The somata of OSNs are located directly at the bases of the sensilla. Additionally to OSN dendrites, sensilla also contain a thecogen cell, a trichogen cell and one or two tormogen cells (Shanbhag et al., 2000), which ensheath the OSNs and produce the sensillar lymph and OBPs (Park et al., 2000; Shanbhag et al., 2001; Larter et al., 2016; Gomez-Diaz et al., 2018).

### 2.2. The first processing center – The antennal lobe

**FIGURE 3:**
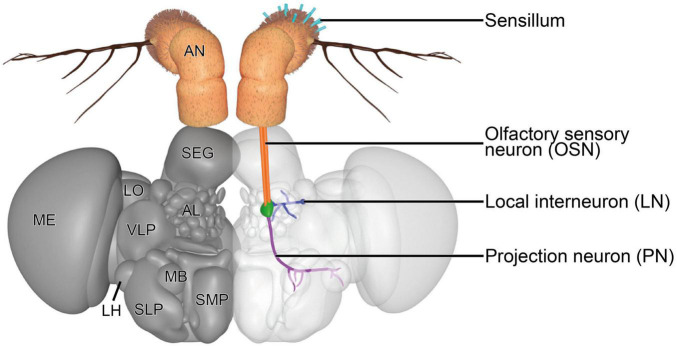
Three-dimensional render of the brain of *D. melanogaster*. A representative olfactory circuit is shown. It consists of OSNs (orange) that innervate a specific type of sensilla on the antenna (teal) with their dendrites and a specific glomerulus (green) with their axons. In the glomerulus, OSN axons synapse onto PNs (violet) and LNs (blue). PNs extend their axons to the MB and LH. LNs usually innervate a diverse set of glomeruli to contact OSNs, PNs, and other LNs, mainly to modulate signal transduction. AL, antennal lobe; AN, antenna; LH, lateral horn; LN, local interneuron; LO, lobula; MB, mushroom body; ME, medulla; OSN, olfactory sensory neuron; PN, projection neuron; SEG, subesophageal ganglion; SLP, superior lateral protocerebrum; SMP, superior medial protocerebrum; VLP, ventrolateral protocerebrum. The 3D model is based on published figures (Jenett et al., 2012; Scheffer et al., 2020) and the brain model of NeuroNLP FlyCircuits (Ukani et al., 2016).

The first-order neurons in the olfactory system are the OSNs. OSNs that innervate the olfactory sensilla on the antenna and maxillary palps extend their axons to the first processing center, the antennal lobe (AL) (Figure 3), *via* the antennal nerve and labiomaxillary nerve, respectively (de Bruyne et al., 1999). These nerves bundle axons of ∼1,200 antennal and ∼120 maxillary OSNs (Singh and Nayak, 1985; Stocker et al., 1990). The deutocerebral AL forms a spherical structure in each brain hemisphere that is connected with the contralateral side *via* the antennal commissure (Stocker et al., 1983). Each AL consists of ∼54–58 small, mostly more or less spherical structures, the so-called glomeruli (Grabe et al., 2015; Schlegel et al., 2021). OSNs that express the same receptor type and which therefore are functionally equal were thought to converge in the same glomerulus (Gao et al., 2000; Vosshall et al., 2000). However, this simplistic model of segregated innervation was recently challenged by a study that showed evidence for a glomerular convergence of OSNs that co-express different receptor families (Task et al., 2022). Additionally, this observation was also made in the mosquito species *Aedes aegypti* (Linnaeus, 1762), indicating that the insect olfactory system might be more complex than was previously thought (Herre et al., 2022). Most OSN types innervate the same glomerulus on the ipsi- and contralateral brain hemisphere (Stocker et al., 1990; Vosshall et al., 2000; Scott et al., 2001). The size, form and location of each glomerulus are characteristic and form a topographic map of the AL that can be used to easily identify the same glomeruli across different individuals (Laissue et al., 1999; Couto et al., 2005; Fishilevich and Vosshall, 2005; Grabe et al., 2015). The relatively well established morphological segregation of glomeruli in the fly’s AL is accomplished by glia cells, which do not just wrap around the entire AL and around individual glomeruli, but also extend processes into glomeruli to ensheath large neuronal processes or bundles of smaller processes (Oland et al., 2008; Zwarts et al., 2015; Kremer et al., 2017).

Within the glomeruli, OSNs, projection neurons (PNs) and local interneurons (LNs) are the three main neuronal types that form a complex network by synapsing onto each other, among each other and in rare cases also onto themselves (autapses) (Rybak et al., 2016; Horne et al., 2018; Scheffer et al., 2020). OSN axons mainly form synaptic contacts with second-order PNs and LNs. The somata of PNs and LNs are located outside of the AL adjacent to its boundaries in the anterodorsal, lateral, and ventral periphery. These three clusters were previously thought to harbor in total ∼150 PN somata (Jefferis et al., 2001) and ∼200 LN somata (Chou et al., 2010; Seki et al., 2010) in a trophospongium formed by cortex glia cells (Zwarts et al., 2015). A recent and detailed connectome study suggests that there are ∼340 PNs that innervate the AL and supports the previously postulated LN number (Schlegel et al., 2021). LNs are a very diverse group of neurons, which extend a process into the center of the AL and branch from there to innervate different glomeruli. Several types of LNs can be distinguished according to their innervation patterns. There are globally innervating LNs that innervate all or almost all glomeruli, locally innervating LNs that innervate different glomeruli in a specific region of the AL, patchy innervating LNs that innervate approximately half of all glomeruli broadly distributed across the whole AL and LNs that only innervate very few glomeruli. Additionally, there are some LNs that also innervate glomeruli in the contralateral half of the AL and that differ in the innervation density within glomeruli (Chou et al., 2010; Seki et al., 2010). LNs form synapses with OSNs, PNs and other LNs. They are as physiologically diverse as they are morphologically, which is also reflected in their neurotransmitter repertoire. LNs release neuropeptides, glutamate, gamma-aminobutyric acid (GABA) and acetylcholine to either inhibit or excite their postsynaptic partners (Ng et al., 2002; Wilson and Laurent, 2005; Olsen et al., 2007; Shang et al., 2007; Olsen and Wilson, 2008; Root et al., 2008; Ignell et al., 2009; Chou et al., 2010; Seki et al., 2010; Das et al., 2011; Liu and Wilson, 2013). There also seems to be a high variability in LN wiring between different flies (Chou et al., 2010), which adds to the complexity of these neurons. Furthermore, some LNs release a combination of these neurotransmitters or form electrical synapses with PNs (Huang et al., 2010; Yaksi and Wilson, 2010). Their morphological and physiological complexity indicates that they fulfill a diverse set of functions that are still largely unknown. Functions that have previously been demonstrated for LNs include gain control by lateral inhibition (Wilson and Laurent, 2005; Olsen and Wilson, 2008), synergistic mixture interaction mediated by lateral excitation (Das et al., 2017) and a selective inhibitory cross-talk between specific glomeruli (Mohamed et al., 2019), which cause a modulation of the incoming signals from the periphery of the olfactory system.

Projection neurons receive the modulated signal and transfer it to the mushroom body (MB) and the lateral horn (LH) in the protocerebrum *via* three different tracts, the medial AL tract (mALT), mediolateral AL tract (mlALT), and lateral AL tract (lALT) (Stocker et al., 1990). Two groups of PNs can be distinguished according to their morphology. The first group consists of PNs that are excitatory and uniglomerular, meaning that they innervate only one glomerulus (uPNs). These acetylcholine expressing uPNs (Yasuyama and Salvaterra, 1999) have their somata in the anterodorsal and lateral clusters and extend their axons along the mALT or lALT to either the MB calyx and the LH or only the LH (Tanaka et al., 2012). The second group consists of PNs that are inhibitory and innervate multiple glomeruli (mPNs). The cell bodies of these GABA releasing mPNs are located in the ventral cluster (Lai et al., 2008). The mPNs extend their axons along the mlALT and bypass the MB to innervate the LH directly (Jefferis et al., 2007; Okada et al., 2009; Tanaka et al., 2012).

### 2.3. The higher brain centers – The mushroom body and lateral horn

The MB and LH harbor third-order neurons, which receive input from the AL *via* axonal terminals of PNs (Figure 3). The MB is a neuropil that is involved in higher cognitive tasks, such as olfactory learning and memory (Dubnau and Tully, 2001; Heisenberg, 2003; Davis, 2005; Fiala, 2007; Busto et al., 2010), context-dependent odor evaluation (Bräcker et al., 2013) and integration of different sensory modalities, such as olfaction (Heisenberg et al., 1985; Stocker et al., 1990), vision (Barth and Heisenberg, 1997; Vogt et al., 2016; Yagi et al., 2016; Sun et al., 2020), gustation (Kirkhart and Scott, 2015), thermo- (Frank et al., 2015), and hygrosensation (Frank et al., 2017). The input region of the MB is its calyx. The innervating PN axons form boutons that are contacted by dendrites of ∼2,500 MB intrinsic neurons, so-called Kenyon cells (KCs) (Technau and Heisenberg, 1982). KCs form characteristic claw-like structures at dendritic terminals. Each of these “claws” synapses onto one bouton, but each bouton is contacted by several KC “claws,” which forms a so-called microglomerulus (Yasuyama et al., 2002; Leiss et al., 2009; Butcher et al., 2012). This way, a given KC receives input from multiple PNs and each PN gives output to multiple KCs, creating a complex network of synaptic connectivity in the MB calyx (Tanaka et al., 2004; Lin et al., 2007; Murthy et al., 2008; Caron et al., 2013; Gruntman and Turner, 2013). KC axons form a dense tract, the pedunculus, which splits up terminally into three lobe-like structures. In the MB lobes, KCs synapse onto relatively few MB output neurons, the axons of which extend deeper into the brain (Tanaka et al., 2008; Strausfeld et al., 2009; Séjourné et al., 2011). Olfactory learning has the potential to affect the number of microglomeruli that respond to the learned odor, rendering the MB a dynamic neuropil that rearranges itself depending on prior experience (Baltruschat et al., 2021).

The LH is the second protocerebral region that receives input from PNs. It is a neuropil involved in innate behaviors that were thought to be independent of the influence of the MB (Wang et al., 2003; Min et al., 2013). Axonal terminals of excitatory PNs branch in a typical pattern. This innervation pattern is very similar, but not identical, between sister PNs that innervate the same glomerulus in a given fly but also between different animals (Marin et al., 2002; Wong et al., 2002; Jefferis et al., 2007). Additionally, PN morphology in the LH seems to be independent of sensory input, since PN axons develop before their dendrites establish a functional connectivity with OSNs and they develop normally even when sensory input is abolished by amputation of antennae and maxillary palps (Wong et al., 2002; Jefferis et al., 2004; Jefferis and Hummel, 2006). The stereotypy of PN axons allows dividing the LH into functional regions, which receive qualitatively different olfactory input (Jefferis et al., 2007; Strutz et al., 2014; Seki et al., 2017; Das Chakraborty and Sachse, 2021). Interestingly, there seems to be a tendency that axons of different PN classes, which innervate different glomeruli but transmit information with a similar valence, form a relatively high number of axo-axonic connections within the LH (Bates et al., 2020; Huoviala et al., 2020). However, higher brain centers such as the MB and LH do not only receive input from the AL, they also give feedback *via* centrifugal neurons (Stocker et al., 1990; Tanaka et al., 2012). This clear functional distinction between the MB as the learning and memory center and the LH as the center that is responsible for innate behavior was challenged in recent years. There is evidence that also the LH is involved in memory tasks (Dolan et al., 2018) and that the MB affects innate behavior (Bräcker et al., 2013; Lin et al., 2014; Lewis et al., 2015).

Olfactory sensory neurons that express a certain receptor and therefore converge onto the same glomerulus, and their associated PNs that also only innervate the same glomerulus, synapse onto these OSNs and extend their axons into the MB and LH, is what neuroscientists often refer to as a neuronal circuit. A given odorant can bind to several different receptors and activate different circuits at the same time. Furthermore, an odorant plume can consist of different odorant molecules that are also very likely to activate different circuits. The activity of different circuits creates an elaborate spatiotemporal map of odorant responses across the AL glomeruli, the so-called combinatorial code (Galizia et al., 1999; Sachse et al., 1999; Vosshall et al., 2000). Even though the amount of different circuits is limited in the fly’s brain, the amount of different odors that can be encoded this way is seemingly endless. It is the task of higher brain centers to decode this information so that the fly can behave adequately.

The connectivity of neurons that are further downstream of the MB and LH is much more complex than the connectivity of first- and second-order neurons in the olfactory system. It is therefore more difficult to define specific olfactory neurons downstream of the MB and the LH. The increasing integration of different sensory modalities in higher brain areas further adds to the complexity (Thiagarajan and Sachse, 2022). In recent years, however, technological advances allowed the acquisition of large-scale connectome data and detailed 3D reconstructions of neurons and their synaptic connections, specific circuits and even entire neuropils (Horne et al., 2018; Zheng et al., 2018; Dolan et al., 2019; Huoviala et al., 2020; Li et al., 2020; Marin et al., 2020; Scheffer et al., 2020; Schlegel et al., 2021). Such large-scale studies are necessary to understand how olfactory information is processed in higher brain areas and how it is transmitted across the brain to finally reach neuromuscular junctions that translate the information into motor activity.

## 3. Experience-dependent plasticity in the olfactory system of insects (excluding *Drosophila*)

Not surprisingly, much of our understanding of experience-dependent plasticity in insect brains is based on the honey bee olfactory system, as it is one of the most extensively studied insect olfactory systems. Early studies demonstrated that the behavioral changes that take place during the transition from hive workers to foragers were accompanied by structural changes in the brain, particularly volume changes of the AL, its glomeruli, and the MB (Withers et al., 1993; Durst et al., 1994; Winnington et al., 1996). Since this behavioral transition depends on the age of the bees, the morphological changes could be predetermined and not dependent on experience. Fahrbach et al. (1998) showed that the volume of MBs of isolated bees reared in darkness increased with age, supporting the age-dependency. However, foraging intensity was shown to be positively correlated with the volume of the lip and collar region of the MB and with the number of boutons in the lip region (Cabirol et al., 2018). The number of boutons in the lip region was further affected by the complexity of the environment in which the bees were reared (Cabirol et al., 2017). These morphological changes were shown to affect learning performance. Alterations in the social environment (Maleszka et al., 2009) and hive temperature (Groh et al., 2004, 2006) are other factors that influenced the morphological development of the MB, suggesting that its architecture and functionality depend not only on age but also experience. However, the MB is not the only brain region in honey bees that is shaped by experience-dependent plasticity. When young hive workers were artificially converted to precocious foragers, the whole AL and specific glomeruli enlarged, and the animals performed better in associative learning tasks of floral scents in comparison to hive workers of the same age (Sigg et al., 1997; Brown et al., 2004). Glomeruli stimulated to grow by this procedure harbored a greater number of synapses. In addition, several studies investigated the effects of long-term exposure to odorants on the AL functionality and morphology. Honey bees exposed to an odorant while having access to sucrose solution showed an odorant-specific increase in glomerular volume. The affected glomeruli also responded more strongly to the specific odorant, leading to increased appetitive behavior in proboscis extension response experiments. The behavioral and physiological changes were observed not only when honey bees were tested with the odor to which they were exposed, but also when they were tested with perceptually similar odorants (Arenas et al., 2009, 2012). Nevertheless, there is evidence that not all glomeruli in the AL are affected equally by odorant exposure and that the exposure procedure may have an influence. Andrione et al. (2017) found that long-term exposure to odorants caused glomeruli to shrink, and Hourcade et al. (2009) showed that Pavlovian conditioning of an odorant caused an odorant-specific enlargement of glomeruli, whereas their activity was unaffected.

In other eusocial hymenopterans, it was shown that experience can also modulate the morphology of the MB. These modulations were mostly attributed to social experience, which may involve olfactory stimulation. For example, *Pseudomyrmex spinicola* (Emery, 1890) is an acacia ant species with division of labor that is not associated with external morphological differences between individuals performing different tasks. There are individuals that forage mainly on leaves (“leaf-ants”) and individuals that stay mainly on tree trunks and defend the colony (“trunk-ants”). This division of labor is not as pronounced in small colonies, where individual ants frequently switch between these tasks. However, as the colony grows, task specialization of ants increases, affecting brain morphology. The MB volume of trunk-ants repeatedly involved in defensive tasks decreases, whereas an opposite effect was observed in leaf-ants, suggesting that memory and learning may play a greater role in specialized leaf-ants (Amador-Vargas et al., 2015). In another ant species, *Camponotus floridanus* (Buckley, 1866), it was shown that the size of the MB is age- and experience-dependent, similar to honey bees. Ants involved in brood care and foraging had more sensory experience and larger MBs than “idle” individuals that remained in the nest without being active (Gronenberg et al., 1996). In the facultative eusocial sweat bee *Megalopta genalis* Meade-Waldo, 1916, it was shown that the behavioral switch to eusociality was associated with division of labor, which affected MB volume depending on the task an individual was performing (Smith et al., 2010; Jaumann et al., 2019). It is hypothesized that eusociality presents animals with additional cognitive demands, such as recognizing nest mates, a task that often involves odor perception (Lorenzi and d’Ettorre, 2020). It is therefore not surprising that neuronal plasticity was observed in eusocial hymenopterans. However, experience-dependent plasticity was also observed in solitary alkali bees (*Nomia melanderi* Cockerell, 1906). Alkali bees kept under deprived laboratory conditions had smaller mushroom bodies than nesting individuals that moved around a complex environment while foraging. In addition, in alkali bees kept in isolation, the region of the MB that receives olfactory input, the lip, was smaller than in individuals paired with another conspecific, suggesting that even in solitary bees, olfaction has the potential to modulate the olfactory system in a social context (Hagadorn et al., 2021). In another solitary bee, the orchard mason bee *Osmia lignaria* Say, 1837, foraging experience was observed to correlate with increased MB size, which was also independent of age (Withers et al., 2008).

The olfactory system of moths and butterflies was shown to be influenced by experience as well. When males of *Spodoptera littoralis* (Boisduval, 1833) were briefly exposed to the female sex pheromone, they were more attracted to the pheromone for more than 24 h after the exposure (Anderson et al., 2003, 2007). In addition, neurons involved in the detection of the pheromone became sensitized, presumably by upregulating the expression of pheromone-binding proteins (Guerrieri et al., 2012). Interestingly, the same behavioral and physiological effects could be elicited by exposing male moths to the sounds of an insectivorous bat, demonstrating a unique case of experience-dependent plasticity across different sensory modalities (Anton et al., 2011). A subsequent study showed that the exposure also affected the morphology of the AL and MB calyx. Both olfactory and auditory stimuli caused volumetric growth of pheromone processing glomeruli in the macroglomerular complex, whereas exposure to the bat sound also caused growth of another glomerulus that processes plant odors (Anton et al., 2016). The AL of *Polygonia c-album* (Linnaeus, 1758) increased in size when these butterflies were exposed to an odor-rich environment with which they could not physically interact. When olfactory input was eliminated unilaterally by covering one antenna in beeswax, enlargement of the ipsilateral AL was prevented, while the contralateral AL showed reduced growth (Eriksson et al., 2019). Another study investigating *P. c-album* demonstrated that MB volume was positively affected when butterflies were exposed to a rich odorant environment while having the opportunity to physically interact with odor sources. However, this treatment did not result in volumetric changes in the AL (van Dijk et al., 2017). Interestingly, two other butterfly species, *Aglais io* (Linnaeus, 1758) and *Aglais urticae* (Linnaeus, 1758), tested in the same study, were demonstrated not to have their MB affected by the enriched environment. Work on the butterfly *Heliconius hecale* (Fabricius, 1775) showed that even insectary-reared butterflies having access to their host plants and additional pollen sources had smaller mushroom bodies than wild-caught individuals (Montgomery et al., 2016). These results suggest that an odorant-rich environment by itself is not always sufficient to cause morphological changes in the AL or MB, or that an odorant environment that is complex for one species might be an impoverished environment for another.

Morphological differences between swarm-forming locusts in the solitary and gregarious phases are an example for polyphenism, a special form of plasticity. It is known from several locust species that the population density of nymphs and also adults can trigger a behavioral and morphological shift from the solitary to the gregarious phase when a certain threshold is reached (e.g., Simpson et al., 1999; Pocco et al., 2019). Interestingly, this phase change could also be triggered in non-swarming locust species with similar behavioral and morphological consequences (Gotham and Song, 2013). Morphological changes associated with the phase shift are not restricted to the outer appearance of the animals, but are also reflected in their nervous system. For example, gregarious desert locusts [*Schistocerca gregaria* (Forsskål, 1775)] have a proportionally bigger MB calyx but proportionally smaller ALs compared to solitary individuals. This observation may indicate a greater need for primary sensory processing in solitary locusts and a greater need for information integration in higher brain centers in gregarious individuals (Ott and Rogers, 2010). Some studies demonstrated that learning of aversive food odors differs between phases (reviewed in Simões et al., 2016), which may argue for a greater need for information integration in the gregarious phase. However, the experience-dependent plasticity observed in locusts is a special case of plasticity compared to previously described examples. The reason is that the gregarization can be initiated in the nymph. Therefore, the observed changes could be partially predetermined in development rather than purely being experience-dependent. Because both nymphs and imagines can gregarize, the behavioral and morphological changes observed in adult individuals may also depend on the time at which gregarization was initiated. The switch from the solitary to the gregarious phenotype can be initiated within a few hours (Ellis, 1963; Roessingh et al., 1993), whereas the switch from the gregarious to the solitary phenotype is a slow process that requires several days, nymphal stages or even generations to complete (Simpson et al., 1999). Moreover, the gregarious phenotype can be inherited to subsequent generations without the need for them to experience an environment crowded with conspecifics (Miller et al., 2008), suggesting that some predetermined factors are indeed involved in this unique form of plasticity.

## 4. Experience-dependent plasticity in the olfactory system of *D. melanogaster*

The olfactory system of *D. melanogaster* is an ideal model for the investigation of experience-dependent plasticity because of its relative simplicity and the genetic tools that are available. Its composition of strongly segregated circuits and the knowledge about odorant tuning profiles for most olfactory receptors allows specific stimulation and precise investigation of certain parts of the system. Two decades ago (Table 1), the first study that documented a case of experience-dependent plasticity in the olfactory system of *D. melanogaster* was conducted by Devaud et al. (2001). From this early study it is known that exposure of flies to odorants for four subsequent days can have an impact on the morphology of the AL. It was shown that the exposure to benzaldehyde or to isoamyl acetate caused a glomerulus-specific volumetric decrease (benzaldehyde: DM2 and V glomerulus; isoamyl acetate: DM6 glomerulus). The volumetric effect after benzaldehyde exposure was shown to be persistent for at least 7 days after the exposure ended and it was accompanied by a slight trend toward a decrease in the number of synapses in the affected glomeruli. Additionally, flies were less repelled by the odorant they were previously exposed to Devaud et al. (2001). A subsequent study from the same authors demonstrated that the experience-dependent plasticity effects can only be evoked when flies are exposed to odorants during early adult life (Devaud et al., 2003). Since these early studies were published, only a few other publications focused on this topic, some of which reported contradictory results on morphological, physiological and behavioral effects.

**TABLE 1:** Summary of the literature on experience-dependent plasticity in the olfactory system of *Drosophila melanogaster* using long-term odor exposure.

References		Devaud et al., 2001	Devaud et al., 2003	Sachse et al., 2007		Chakraborty et al., 2009	Iyengar et al., 2010	Das et al., 2011	McCann et al., 2011	Sudhakaran et al., 2013	Kidd et al., 2015	Kidd and Lieber, 2016	Golovin et al., 2019		Chodankar et al., 2020		Gugel et al., 2022		Fabian, 2022		
Odors for exposure		Benzal–dehyde, isoamyl acetate	Benzaldehyde, isoamyl acetate	Ethyl butyrate, CO_2_		Ethyl acetate, isoamyl acetate, furfuryl acetate, butanol	Ethyl acetate	Ethyl butyrate, CO_2_	Ethyl butyrate, CO_2_	Ethyl butyrate, CO_2_	Geranyl acetate, CO_2_	Geranyl acetate	Ethyl butyrate		Ethyl butyrate, CO_2_		E2-hexenal, 2-butanone, geranyl acetate		Geosmin, 3-hexanone, ethyl hexanoate		
Glomerulus volume		DM2, DM6, V	DM2, DM6, V	DM2, V				DM2, DM5, DM6, V	DM2, DM5, V		V, VA6	VA6	VM7d		DM2, DM5, V	VM7d			DA2, DM1, DM2		
Glomerulus-specificity																					
Cellular effects	OSNs																				
	PNs																				
	LNs																				
Physiology	OSNs			GCaMP		SSR	Extracellular single-unit recordings				GCaMP						Patch-clamp		GCaMP		
	PNs			GCaMP				GCaMP	GCaMP	GCaMP	GCaMP	GCaMP					PC	PC	GCaMP		
	LNs			GC	GC																
Behavior		T-maze	T-maze	Walking		Trap		Y-maze	Y-maze	Y-maze	Trap				Y-maze				T-maze, oviposition		
Reversibility																					
Early critical period																					
Involved genes, neurotransmitters, and signaling pathways		*Dunce*	*Dunce, rutabaga*					*Rutabaga*, GABA, NMDAR1	Ataxin-2	Ataxin-2, dFMR1, CaMKII	*Notch, disabled*	*Delta*	NMDAR1		*Rutabaga*						

Colored tiles indicate experiments that were done in the respective studies. Green, a phenomenon was observed (e.g., glomerulus-specificity) or there was an increase in the measured parameter (e.g., glomerulus volume); orange, a phenomenon was not observed (e.g., reversibility) or there was a decrease in the measured parameter (e.g., physiology); gray, the measured parameter was unchanged. GC, GCaMP; LNs, local interneurons; OSNs, olfactory sensory neurons; PC, patch-clamp; PNs, projection neurons.

By focusing on other neuronal circuits, it was shown several years ago that the exposure to carbon dioxide or ethyl butyrate affects the associated V and DM2 glomerulus, respectively (Sachse et al., 2007). However, contrary to the mentioned previous studies, the glomeruli grew in size instead of shrinking due to the exposure. This finding was the first indicator that not every olfactory circuit is affected in the same way by the perception of odorants. Furthermore, it could be demonstrated that two subsequent days of odorant exposure are sufficient to induce the maximum volumetric effect in the V glomerulus. When flies were returned to ambient air for at least 2 days after the exposure period, the glomerulus size returned to normal. This observation of reversibility differs from the observations of Devaud et al. (2001). Nevertheless, the existence of an early critical period for the induction of experience-dependent plasticity effects was supported, as glomerular growth could not be induced when flies were exposed relatively late in their adult life (Sachse et al., 2007). Behavioral experiments demonstrated that carbon dioxide-exposed flies respond less to the odorant in walking assays, which is in line with Devaud’s findings as well. To elucidate the cellular processes that are involved in glomerulus volume changes, the OSNs that innervate the V glomerulus were investigated more precisely (Sachse et al., 2007). Quantifications of OSN cell somata in the antenna did not reveal any effects on OSN number due to odorant exposure. Moreover, detailed 3D reconstructions of OSN axonal terminals inside the V glomerulus and quantification of the number of their terminals, their length and volume showed that they were not affected by exposure to carbon dioxide either. These findings indicate that glomerular growth does not depend on changes in OSN number or morphology. Physiological experiments demonstrated that the signaling of OSNs in carbon dioxide-exposed flies was not changed. However, the output signaling of PNs in the LH was lower in exposed flies and this decrease could be explained by increased activity of inhibitory LNs (Sachse et al., 2007). Two follow-up studies by Das et al. (2011) and McCann et al. (2011) targeted the DM2, DM5, and V glomeruli and confirmed the morphological, physiological, and behavioral plasticity effects.

When flies are exposed to geranyl acetate, the associated VA6 glomerulus grows in size as well, which is an effect that was shown to be reversible (Kidd et al., 2015; Kidd and Lieber, 2016). The volumetric growth does not seem to rely on an early critical period, as it could also be evoked in flies that were exposed beginning on the eighth day after hatching from the pupa (Kidd and Lieber, 2016). Interestingly, exposure to geranyl acetate increases the attraction toward this odor in trap assays, which contradicts previous findings that showed that exposure to odors decreases the behavioral response toward them. The authors hypothesized that flies could form a positive association between the odorant and food during the exposure time, which could increase the attractiveness of the odor. Such a positive association might only occur when flies are exposed to neutral or attractive odorants such as geranyl acetate and not when they are exposed to repellent odors such as carbon dioxide or benzaldehyde. Additionally, exposure to geranyl acetate decreases responses of associated OSNs and increases PN responses (Kidd et al., 2015). Similarly, exposure to the neutral or attractive odors ethyl acetate, isoamyl acetate, furfuryl acetate or butanol (Knaden et al., 2012) was shown to cause an increased attraction toward the odor that was used during the exposure period (Chakraborty et al., 2009). This observation was correlated with an increased spike rate of different OSN classes. Moreover, OSNs of flies that were reared on a medium that contained ethyl acetate but was otherwise odorless showed an increased sensitivity to low ethyl acetate concentrations afterward (Iyengar et al., 2010).

Recent studies documented some additional interesting plasticity effects in the AL. Golovin et al. (2019), exposed flies to ethyl butyrate and investigated the consequences for the VM7d glomerulus (called “VM7” by the authors). The authors indicate to have observed a volumetric decrease of the VM7d glomerulus after flies were exposed to ethyl butyrate (Golovin et al., 2019; Figure 1). But what they actually measured according to the description of their methods was only GFP positive voxels of the OSN labeling, indicating that the OSNs retract their terminals from the area of the VM7d glomerulus. This finding is of particular interest because it is contradictory to the findings for OSNs of the V glomerulus, which were unchanged after flies were exposed to carbon dioxide (Sachse et al., 2007). The retraction of OSN axons was accompanied by a decrease of synaptic area and number of T-bars. Information about the actual glomerulus volume is lacking. Even though Golovin et al. (2019) emphasized on the presence of an early critical period for experience-dependent plasticity effects, they demonstrate that very high odorant concentrations can still induce plasticity effects relatively late in the adult life (7–9 days after flies hatched from the pupa). This observation may indicate that the ability to undergo plastic changes might be maintained throughout adulthood, or at least longer than previously thought, and that only the conditions to evoke them change. The reversibility of plasticity effects was also observed in the VM7d circuit, as was an unchanged number of OSNs, which is in line with previous observations (Sachse et al., 2007).

A subsequent study by Chodankar et al. (2020) followed up on Golovin et al. (2019). They support that the OSN axons that innervate the VM7d glomerulus (called “VM7” by the authors) seem to retract during long-term exposure to ethyl butyrate. The OSNs rapidly recover within 12 h when flies are supplied with ambient air after the exposure period. However, they demonstrate that the glomerular volume of VM7d is not affected by the exposure. In an experiment in which the authors exposed flies to ethyl butyrate or carbon dioxide and subsequently measured GFP positive pixels of PN labeling in associated glomeruli, they found that the PN dendrites increase in size in a glomerulus-specific manner. This finding reveals the growth of PN dendrites as one factor that contributes to the glomerular growth. Moreover, the authors support that plasticity effects in the VA6 glomerulus are independent of an early critical period, as this glomerulus also grows in size when flies are exposed to geranyl acetate relatively late in adulthood. Contrary to the VA6 glomerulus, the critical periods of the DM5 and V glomeruli close within 48 h after eclosion, after which the induction of glomerular growth is no longer possible (Chodankar et al., 2020).

For the publications mentioned so far, which dealt with experience-dependent plasticity in the olfactory system of *D. melanogaster*, very high odorant concentrations were used over an exposure period of usually four subsequent days (Golovin et al., 2019: only 2 days). In these studies, odorant supply was in most cases realized by a perforated centrifuge tube containing the odorant solution, which was placed inside a container that contained flies. Therefore, the odorant molecules were omnipresent throughout the exposure period, resulting in intense and unnatural stimulation of receptors (Gugel et al., 2022). At such high concentrations, the binding of odorant molecules to receptors is less specific (Hallem and Carlson, 2006; Hu et al., 2020), which could lead to combinatorial effects that are difficult to interpret and to detect as such. Moreover, due to these high concentrations and intense exposure procedures, observed exposure effects could be caused by excitotoxicity instead of experience-dependent plasticity. In order to prevent such unpredictable experimental influences, Gugel et al. (2022) used a pulsed exposure system with 1 s of odor at relatively low concentrations followed by 20 s ambient air. A similar pulsed exposure was already utilized previously (Sachse et al., 2007) but only for a single experiment. This exposure method allows flies to be exposed in a more subtle manner that also more closely resembles natural conditions, where a particular odor is usually not present all the time. Another positive side effect of a pulsed exposure system is that neuronal responses are less likely to adapt to the exposed odor (Gugel et al., 2022). Gugel and colleagues exposed flies to E2-hexenal, 2-butanone or geranyl acetate at concentrations that specifically stimulate OSNs that converge in the DL5, VM7d (called “VM7” by the authors), and VA6 glomerulus, respectively. With physiology experiments, they could demonstrate that OSN signaling is unaffected by the exposure procedure. However, the responses of DL5 and VM7d PNs to low odorant concentrations were increased when flies were exposed to either E2-hexenal or 2-butanone. The same effect was not observed in VA6 PNs when flies were exposed to geranyl acetate, demonstrating another odorant- or glomerulus-specific effect. Experiments showed that the excitability of PNs and the synaptic strength between OSNs and PNs does not explain the increased PN response for low odorant concentrations as both of these factors were unaffected by the exposure. Gugel et al. (2022) found that odor-evoked lateral excitation could be the reason for increased PN responses. Interesting observations in this study were glomerulus non-specific effects, which contradict previous publications that emphasized odor- and glomerulus-specificity of experience-dependent plasticity (Sachse et al., 2007; Das et al., 2011; Kidd et al., 2015; Kidd and Lieber, 2016; Chodankar et al., 2020). One of these non-specific effects was an increased spontaneous firing rate of VM7d PNs, although the flies were only exposed to E2-hexenal (which stimulates the DL5 glomerulus) and not 2-butanone. The exposure to E2-hexenal also caused a more prominent and prolonged hyperpolarization in VM7d PNs, which should not be affected by exposure to the odorant if the effects were purely glomerulus-specific. Morphological investigations revealed that exposure to E2-hexenal caused a variety of different glomeruli to be slightly smaller, an effect that was only significant when compared across all measured glomeruli. Additionally, the innervation density of NP3056 LNs in E2-hexenal-exposed flies was slightly higher in all measured glomeruli. This effect was also only significant when compared across different glomeruli. Those morphological effects were not observed when flies were exposed to 2-butanone.

In a recent study, we asked whether even a highly specific olfactory circuit, which is crucial for the survival of *D. melanogaster* because it conveys information about the presence of potentially toxic microorganisms (Stensmyr et al., 2012) is subject to plasticity as well (Fabian, 2022). For this, flies were exposed to geosmin in a pulsed way similarly to Gugel et al. (2022), so that flies perceived the odor for 1 min and subsequently ambient air for 5 min. As expected, the exposure caused a specific increase in volume of the geosmin-responsive DA2 glomerulus. We could reveal some of the first cellular effects that are associated with the glomerular growth. The dendrites of innervating PNs expanded in response to geosmin-exposure and clearly contributed to the volumetric growth of the glomerulus, which supports the observations of Chodankar et al. (2020). We also observed bigger PN somata in geosmin-exposed flies, possibly as a response to higher metabolic demands of larger dendrites. Single-cell photoactivation of different LN subpopulations revealed glomerulus-specific morphological changes at the level of boutons and terminals in globally innervating LNs, while patchy LNs were unaffected. These findings show that also LNs undergo major structural rearrangements in response to long-term odor exposure.

Notably, OSN axons and glia cell processes extending into the DA2 glomerulus were shown to not contribute to the glomerular growth (Fabian, 2022). However, the fission of mitochondria in OSNs innervating the DA2 glomerulus was the first evidence of morphological changes of subcellular structures caused by long-term odorant exposure. Despite structural rearrangements in PNs and LNs, there were no significant changes in the neuronal activity of OSNs and PNs innervating the DA2 glomerulus, which is in line with observations by Gugel et al. (2022). The observed reduced behavioral response to geosmin after flies were exposed to the odorant could be caused by physiological changes in higher brain centers (Gugel et al., 2022).

In contrast to work examining experience-dependent plasticity in the MB or LH in other insects, there are only few studies linking structural and functional changes in these higher olfactory brain centers of *D. melanogaster*. Disruption of signaling in PNs was shown to affect the physiology and morphology of microglomeruli in the MB (Kremer et al., 2010; Pech et al., 2015; Doll et al., 2017), suggesting that experience-dependent plasticity is also a feature of olfactory neuropils other than the AL. Similarly, silencing OSNs affects the morphology of axonal terminals of PNs in the MB (Figure 3 in Hayashi et al., 2022). However, not only the inability to experience odors evokes plasticity effects in the MB, but also the sensation of odorants during appetitive learning. It was shown that the formation of long-term appetitive memory for the pheromone 11-cis-vaccenyl acetate is associated with an increased number of functioning microglomeruli formed by PNs involved in the sensation of this compound (Baltruschat et al., 2021). Whether the LH is subject to experience-dependent plasticity is largely unknown. We performed the first experiments examining the influence of prolonged odor exposure on the branching patterns of PN axons in the LH and found that it remained unchanged (Fabian, 2022). Further studies are needed to uncover and understand experience-dependent plasticity effects in the MB and LH more broadly. These studies face the challenge of disentangling the influence of olfaction from other sensory modalities that converge in these neuropils.

## 5. Molecular underpinnings of experience-dependent plasticity in the antennal lobe of *D. melanogaster*

For many of the morphological, physiological and behavioral consequences of long-term exposure to odorants, molecular and genetic drivers were found (Table 1). Early studies discovered that flies with a mutation in the *dunce* gene (*dnc*^1^) do not exhibit changes in glomerular volumes or behavior in response to odorant exposure (Devaud et al., 2001, 2003). This gene encodes a phosphodiesterase that is responsible for the degradation of cyclic adenosine monophosphate (cAMP). Consequently, a mutation in this gene leads to increased levels of cAMP in the cell (Davis and Kiger, 1981; Scheunemann et al., 2018), which has the potential to detrimentally affect intracellular signaling (Lee, 2015). These findings indicate that the intracellular cAMP level seems to be responsible for glomerular volume changes and behavioral changes (Devaud et al., 2001). Similarly, glomerular volumes, neural physiology and behavior in *rutabaga* (*rut*^2080^) mutants are unaffected by long-term odor exposure (Devaud et al., 2003; Das et al., 2011). The *rutabaga* gene encodes a Ca^2+^/calmodulin-responsive adenylate cyclase, which is involved in the synthesis of cAMP (Livingstone et al., 1984; Levin et al., 1992). A mutation in this gene causes decreased levels of intracellular cAMP, further highlighting the importance of cAMP for experience-dependent plasticity effects. Das et al. (2011) could demonstrate that the activity of *rutabaga* is particularly important in *NP1227-Gal4* (LN1) LNs for the formation of short- and long-term behavioral habituation caused by long-term odor exposure. Additionally, the enhanced branching of PN dendrites that coincides with glomerular growth during odorant exposure was inhibited in *rut*^2080^ mutants or when *rutabaga* was knocked down in *NP1227-Gal4* LNs. The effect could be rescued in *rut*^2080^ mutants when functional *rutabaga* was specifically expressed in *NP1227-Gal4* LNs (Chodankar et al., 2020). Das et al. (2011) found that artificial expression of an inhibitory form of the cAMP response element-binding protein (CREB) in *NP1227-Gal4* LNs blocked experience-dependent behavioral and morphological changes as well, highlighting the importance of cAMP signaling for plasticity effects in the AL. Moreover, dFMR1 and Ataxin-2 (Atx2), RNA-binding proteins that have been found to be involved in the repression of translation by microRNAs, were shown to be important for the establishment of plasticity effects as well (McCann et al., 2011; Sudhakaran et al., 2013). For example, a knockdown of Atx2 in PNs abolishes morphological, behavioral and physiological effects that occur after long-term exposure of flies to carbon dioxide or ethyl butyrate.

Another gene that was reported to play a role in experience-dependent plasticity in the olfactory system of *D. melanogaster* is *notch* (Kidd et al., 2015; Kidd and Lieber, 2016). This gene encodes a transmembrane receptor protein (Wharton et al., 1985). It is involved in signal transduction between cells and fulfills various functions during developmental processes (e.g., Brückner et al., 2000; Ramain et al., 2001; Okajima et al., 2003; Wilkin et al., 2004). Knocking down *notch* in V or VA6 OSNs abrogates the glomerular growth that is induced by long-term exposure to carbon dioxide or geranyl acetate, respectively. The knockdown of *notch* in OSNs also abolishes the physiological effects of odorant exposure that the authors observed in OSNs and PNs. Furthermore, overexpression of the adaptor protein Disabled (Dab), which physically associates with transmembrane receptors, blocks the growth of the VA6 glomerulus in geranyl acetate-exposed flies (Kidd et al., 2015).

The gene *delta* encodes a ligand for the notch receptor (Rebay et al., 1991) and it is therefore not surprising that the manipulation of *delta* has an impact on odor-evoked plasticity effects as well. The increased neuronal response of VA6 PNs that were observed in geranyl acetate-exposed flies is abolished when *delta* is knocked down in these PNs (Kidd and Lieber, 2016). Surprisingly, this knockdown causes an amplified volumetric growth of the glomerulus in odor-exposed flies. These findings indicate that the relationship between physiological and morphological plasticity effects is not straightforward. The authors conclude that the notch pathway affects the glomerular plasticity by canonical and non-canonical mechanisms. Interestingly, the VA6 glomerulus still grows in size when vesicle release in the associated OSNs is blocked by tetanus toxin, indicating that the volume increase is independent of activation of downstream neurons. The authors hypothesize that the *delta* pathway could be activated by neuropeptide signals from OSNs to PNs. Moreover, the notch pathway was shown to be involved in the reversibility of the plasticity effect. When *delta* is knocked down, the volumetric increase of glomeruli that is caused by exposure to odorants is irreversible for 2 days after the exposure (Kidd and Lieber, 2016).

In the context of retracting OSN axons and the associated decrease of synaptic area and T-bars in the VM7d glomerulus, the Wnt signaling pathways were investigated in more detail (Golovin et al., 2019). Wnt signaling is involved in many processes, including the regulation of synaptic connections between neurons (Packard et al., 2002; Chiang et al., 2009; Korkut et al., 2009; Friedman et al., 2013; Kopke et al., 2017). Therefore, this kind of signaling could be involved in the morphological effects observed in this study after flies were exposed to ethyl butyrate. However, perturbations at different steps of the Wnt signaling pathway revealed that the morphological alterations are independent of this kind of signal transduction (Golovin et al., 2019).

Beside the experiments that aimed at different gene regulatory networks, some studies focused on synaptic transmitters in order to elucidate underlying mechanisms of experience-dependent plasticity. For example, Das et al. (2011) targeted the signaling between LNs and PNs. They demonstrated that a reduction of the synaptic output of GABAergic *NP1227-Gal4* LNs inhibits the behavioral habituation and morphological effects of long-term odor exposure. In contrast, artificial activation of these LNs was sufficient to elicit the plasticity effects. The knockdown of GABA_*A*_ receptors in PNs blocks the GABAergic signaling between LNs and PNs and likewise abolished behavioral but not morphological plasticity effects. Similarly, the inhibition of glutamate signaling from LNs to PNs by knockdown of the NMDAR1 receptor in PNs was shown to block the behavioral habituation and prevent the glomerular growth. These findings demonstrate that both glutamatergic and GABAergic signaling are involved in experience-dependent plasticity effects in the AL.

Golovin et al. (2019) knocked the GABA_*B*_ receptor in VM7d OSNs down, which had no effect on the retraction of OSN axons that they observed after exposing flies to ethyl butyrate. However, when they knocked down the glutamatergic NMDAR1 receptor specifically in VM7d OSNs, they could not observe an experience-dependent effect on the OSN axons. Therefore, for experience-dependent plasticity effects, the glutamate signaling seems to play a role in PNs (Das et al., 2011) as well as OSNs (Golovin et al., 2019). Blocking the neurotransmission to downstream neurons by expressing tetanus toxin in VM7d OSNs, did not inhibit the retraction of their axons but even increased the effect that odorant exposure has on these neurons. Interestingly, when the authors abolished action potentials in VM7d OSNs by expressing the exogenous inward rectifying potassium channel 2.1 (Kir2.1) in these neurons, OSN axons did not just retract in odorant-exposed flies but also in flies that were only exposed to the vehicle control. The authors argue that neuronal activity does not just seem to be needed for the control of OSN refinements, but also for the initial innervation of the VM7d glomerulus (Golovin et al., 2019). A similar experiment by Chodankar et al. (2020) could show that the critical period for odor evoked plasticity is extended in flies in which OSNs were silenced with Kir2.1 for the first 2 days after eclosion.

All these genetic and molecular findings demonstrate that experience-dependent plasticity in the AL underlies various signaling pathways with complex interactions (reviewed in Golovin and Broadie, 2016). Moreover, the aforementioned molecular mechanisms are not only involved in the generation of experience-dependent plasticity effects, but also in other cellular processes, adding to the complexity. From the limited data available, it is impossible to conclude whether some of these molecular mechanisms occur in a general manner across different glomeruli and circuits. Future studies need to uncover glomerulus-specific and more general principles to understand how plasticity effects arise in different olfactory circuits of the AL.

## 6. Limiting factors for the investigation of experience-dependent plasticity effects

The investigation of long-term plasticity effects in the olfactory system of insects poses a challenge. We have to assume that some individuals are strongly affected by the exposure procedure while others might not be affected at all, without any evidence to indicate which of the exposed flies were not affected. For example, in our recent study, we observed that the standard deviation of the volume of the DA2 glomerulus was in most cases higher in the group of animals that perceived geosmin (Fabian, 2022) compared to the control group receiving the solvent control mineral oil, indicating an increased variability in the experimental group. This could, of course, conceal very subtle morphological effects. The difficulty of detecting activity-dependent effects in neuronal circuits within inter-animal variability is a serious issue that was also mentioned in a study on plasticity effects of MB input synapses (Kremer et al., 2010). Golovin et al. (2019) suggest that the onset of odor exposure very early, at the late pupal stage, leads to more robust phenotypes, which could lower inter-animal variability. This approach should be considered for future experience-dependent plasticity studies to increase the chances of detecting very subtle plasticity effects. In addition, the introduction of methods to study morphological parameters in the olfactory system before and after exposure would allow us to document subtle changes within individuals. But, of course, such experiments are challenging to perform and involve additional difficulties concerning the ethical treatment of animals.

The measurement of morphological characters only at specific time points during the development also poses a problem. Just because no morphological changes were observed in the neurites of neurons after the exposure period it does not necessarily mean that there were no changes at all. During development, OSNs axons, for example, frequently extend and retract their axons (Li et al., 2021). In the developing larval visual system, these extensions and retractions of neurite terminals were shown to occur at the same rate, so that the overall length did not change (Sheng et al., 2018), although intense dynamic processes were at work. However, such dynamic plasticity effects could only be detected in adult flies if glomerular growth could be observed in real time. Moreover, the glomerular growth was shown to be reversible in some olfactory circuits (Sachse et al., 2007; Das et al., 2011; Kidd et al., 2015), emphasizing the importance of the time point at which experiments are conducted. Kidd et al. (2015) suggested that differences in the exposure paradigm, e.g., the time window between the end of the exposure and the beginning of the experiment, could explain the discrepancy in the results of some studies. Morphological results show that at least PNs and LNs undergo extensive structural changes (Chodankar et al., 2020; Fabian, 2022) during the exposure period and that in one olfactory circuit also OSN axons were affected (Golovin et al., 2019; Chodankar et al., 2020). It is likely that synapses are not yet properly established in the grown glomerulus, which could lead to different responses at different time points after the end of the exposure period because the measurements are taken in the middle of an ongoing developmental process.

There is evidence that the observed volumetric effects could also be influenced by methodological procedures. Specific changes in glomerular volume were consistently observed in all of our *in vivo* experiments but, surprisingly, not in *in vitro* experiments (Fabian, 2022). In addition, the DA2 glomerulus was strikingly smaller in the *in vitro* experiments than in the *in vivo* experiments. The dissection and fixation of brain tissue adversely affects its volumetric properties (Ma et al., 2008; Grabe et al., 2015), which could conceal subtle morphological differences in some cases.

## 7. Discussion

All of the mentioned observations in the olfactory system of *D. melanogaster* and other insect species draw a clear picture – plasticity effects in this part of the nervous system are common in insects. In general, neuronal plasticity is also known from other arthropods, such as crustaceans (Harrison et al., 2002; Sandeman and Sandeman, 2003) and jumping spiders (Steinhoff et al., 2018), but also in other invertebrates like cephalopods (Dickel et al., 2013) and gastropods (Bailey and Chen, 1991; Bailey and Kandel, 2008). It is therefore likely that neuronal plasticity is a widespread feature of centralized nervous systems.

Most studies that focused on plasticity effects in the olfactory system of insect species observed volumetric changes in entire neuropils like the MB and AL. Only a few studies managed to resolve plasticity effects on the scale of individual glomeruli. These morphological changes may be triggered, for example, by foraging, social, or olfactory experiences. In species other than *D. melanogaster* it is more difficult to link morphological changes to functional changes. However, as genetic techniques for generating genetically modified strains advance, we are likely to obtain more detailed data in the future for comparison with observations in *D. melanogaster*.

The knowledge we already have today about experience-dependent plasticity in the olfactory system of *D. melanogaster* indicates that exposure to odorants can cause a variety of opposing morphological, physiological and behavioral effects. These effects are likely to depend on the odorants that were used and the glomeruli that were investigated. Although only very few odorants and glomeruli were investigated in the context of experience-dependent plasticity so far, there was no general effect found that applies to all tested combinations of glomeruli and odorants (Table 1). Some studies report decreased, increased, or unchanged glomerular volumes after flies were exposed to certain odorants. If glomerular sizes are affected, these effects can either be long-lasting or reversible, glomerulus-specific or general, and dependent or independent of an early critical period. The physiological response of associated neurons is also either higher, lower, or unaffected. Similarly, the behavioral response to the exposed odorant can also be either enhanced or weakened dependent on which odorant flies were exposed to during the exposure period. But why are such opposing effects observed across different studies? An obvious answer to this question could be that various circuits in the olfactory system underlie some different molecular mechanisms that cause these circuit-specific effects. Additionally, the impact of the odorant and the resulting changes in the olfactory system could depend on its hedonic valence, which might be specifically important for the behavioral effects of long-term exposure. However, methodological issues cannot be excluded. Although the exposure method is similar in a few studies, it still varies at least in some fine details, which could influence the outcome of the experiments. Standardized procedures in future investigations would certainly help to gather data that is more comparable between studies.

Most mentioned studies that investigated plasticity effects caused by long-term odorant exposure in *D. melanogaster* used extremely artificial exposure procedures. A continuous exposure period of 2–4 days with odorant concentrations between 0.1% and 25% (Devaud et al., 2001, 2003; Sachse et al., 2007; Das et al., 2011; Kidd et al., 2015; Kidd and Lieber, 2016; Golovin et al., 2019; Chodankar et al., 2020) is very intense and unlikely to occur under natural conditions. At high odorant concentrations, it is more likely that many OSN classes are stimulated, making interpretations of the results difficult (Gugel et al., 2022). The first study to attempt to mimic natural conditions by using much lower odorant concentrations and a periodic exposure method was conducted by Gugel et al. (2022). Such less intense exposure procedures resemble natural conditions more closely. Nevertheless, because glomerular growth was observed in the laboratory under admittedly high odorant concentrations, it is conceivable that it must also occur under natural conditions, since such structural changes are costly and the underlying mechanisms that induce these changes should not become established over evolutionary time periods if they were meaningless. Most olfactory circuits are broadly tuned and activated by many different odorants (Hallem and Carlson, 2006). Therefore, it is conceivable that it is not necessary to expose a fly to high concentrations of a single odorant over a long period of time to induce changes in broadly tuned circuits. Exposure to varying concentrations of different odorants that bind to the same receptor, thereby activating the same underlying neural circuit, might be sufficient to elicit the same plasticity effects that were observed after exposure to a single highly concentrated odorant. Such complex and dynamic stimulation is more likely to occur under natural conditions (Gorur-Shandilya et al., 2019). It would be interesting to see if future studies could replicate the same plasticity effects in broadly tuned circuits using different odors at fluctuating concentrations during the exposure period. To better understand how the observed plasticity effects might occur under natural conditions, it would also be worthwhile to investigate which is the least intense exposure procedure (exposure duration, odor concentration, and stimulus frequency) that still elicits the effects.

Highly specific receptors such as Or56a, GR21a, GR63a or pheromone receptors are crucial for an animal’s behavior. At first glance, it seems puzzling that circuits with a high specificity like the geosmin circuit, which conveys information about the presence of toxic threats (Stensmyr et al., 2012), change based on past experience. One reason for observing experience-dependency in such highly specific olfactory circuits could be their evolutionary past. It is likely that these circuits did not emerge *de novo*, but evolved from a more broadly tuned circuit and became more specific over time. For example, the emergence of new olfactory circuits could be caused by gene duplications (Prieto-Godino et al., 2017) or by suppression of apoptotic events during development (Prieto-Godino et al., 2020). Therefore, it is credible that narrowly tuned and broadly tuned circuits share at least some similar molecular mechanisms that cause experience-dependent plasticity effects. However, the ability to exhibit plastic changes in highly specific circuits may be more than just a relic of their evolutionary past. Plastic effects in neuronal tissue are an efficient way to adjust the energetically expensive nervous system in response to changing environmental conditions (Eriksson et al., 2019) to optimize the cost-benefit ratio (Niven and Laughlin, 2008).

Moreover, plasticity effects could also play a role in speciation processes. The data show that a female fly exposed to an aversive odorant such as geosmin for an extended period of time subsequently behaves indifferently to the odorant. It is likely to lay more eggs on a medium exposed to a geosmin source, and thus the offspring is exposed to the odorant throughout its development. Golovin et al. (2019) suggest that under such circumstances, the exposure-related phenotype is more robust. If several subsequent generations are exposed to the odor, it could become meaningless for that subpopulation. Under such conditions, random mutations in genes involved in the development of a particular olfactory circuit could accumulate because there would be a lack of positive selection pressure acting upon the integrity of the associated gene-regulatory network. Eventually, the olfactory circuit might cease to function, leading to distinctive behaviors in a genetically segregated subpopulation – a potential starting point for speciation processes. However, the consequences do not necessarily have to be so drastic that an olfactory circuit becomes useless. There could also be much more subtle effects, such as changes in the specificity and sensitivity of OSNs. For example, shifts in host plant use by distinct subpopulations of *Drosophila mojavensis* Patterson and Crow, 1940 have been shown to be associated with changes in their olfactory systems (Crowley-Gall et al., 2016), which are also involved in reproductive isolation between subpopulations (Khallaf et al., 2020).

Interesting observations were made in studies in which *D. melanogaster* cohabitated with the parasitoid wasp *Leptopilina heterotoma* (Thomson, 1862). Flies exposed to these wasps developed a preference for ethanol-containing substrate for oviposition, as an ethanol-rich diet protected larvae from parasitoids (Kacsoh et al., 2013). Surprisingly, this behavioral change was observed in subsequent generations, although the offspring never experienced the presence of wasps (Bozler et al., 2019). Furthermore, exposure of subsequent fly generations to wasps enhanced the effect. To date, it is not known which stimulus triggers the transgenerational effect. However, it is likely that the sense of smell is involved, since *L. heterotoma* produces iridomyrmecin, a compound that activates a dedicated, highly specific olfactory circuit in *D. melanogaster* (Ebrahim et al., 2015). Elucidating plasticity effects in this olfactory circuit might be worthwhile to investigate in future studies. Whether exposure to other odorants also has the potential to elicit transgenerational plasticity effects that may be enhanced by exposure of multiple subsequent generations is another intriguing question.

Experience-dependent plasticity in the insect olfactory system as a consequence of an altered chemical environment has the potential to change odorant perception and associated behavior over relatively short time scales. Similar to associative learning (Cook et al., 2020), it may contribute to insect resilience to the effects of climate change and environmental pollution and therefore remains an important area of research for the future.

## Author contributions

BF and SS conceived and wrote the manuscript. Both authors contributed to the article and approved the submitted version.
